# Typing of *Staphylococcus aureus* obtained from mastitic milk of cattle and buffalo on the basis of two virulence-associated genes (*spa and clf*A) 

**DOI:** 10.14202/vetworld.2015.398-402

**Published:** 2015-03-26

**Authors:** Rahul Yadav, Sandeep Kumar Sharma, Jyotika Yadav, Anil Kumar Kataria

**Affiliations:** 1 Department of Veterinary Microbiology and Biotechnology, College of Veterinary and Animal Sciences, Rajasthan University of Veterinary and Animal science, Bikaner, Rajasthan, India; 2 Department of Veterinary Microbiology and Biotechnology, Post Graduate Institute of Veterinary Education and Research, Rajasthan University of Veterinary and Animal Science, Jaipur, Rajasthan, India; 3 College of Veterinary and Animal Sciences, Hisar, Lala Lajpat Rai University of Veterinary & Animal Sciences, Hisar, Haryana, India

**Keywords:** buffalo, cattle, *clf*A gene, mastitis, *Staphylococcus aureus*, *spa* (X-region) gene

## Abstract

**Aim:**

The present study was undertaken to type *Staphylococcus aureus* isolates from cattle and buffalo mastitic milk on the basis of *spa* (X-region) and *clf*A genes, both responsible for producing virulence factors.

**Material and Methods:**

In the present investigation *S. aureus* isolates were isolated as per standard protocols. Typing of *S. aureus* was carried out by molecular detection of *spa* and *clf*A gene by polymerase chain reaction.

**Results:**

All the 32 isolates from cattle (16) and buffalo (16) were divisible into seven *spa* types with amplicon sizes ranging between 120 and 380bp. The cattle isolates produced seven different *spa* amplicons of 120, 150, 200, 250, 280, 300, and 330 bp with 3, 4, 6, 8, 10, 11 and 12 number of tandem repeats, respectively whereas buffalo isolates were divisible into five *spa* types with amplicons of 150, 200, 250, 330 and 380 bp having calculated number of repeats of 5, 7, 9, 12, and 14, respectively. Of the total isolates, 24 were considered pathogenic on the basis of more than seven number of tandem repeats. In the present investigation, *clf*A gene was amplified in 27 isolates from cattle and buffalo producing two different amplicons of 900 and 1000 bp sizes showing polymorphism. The most (71.80%) of the isolates produced amplicons of 900 bp while amplicon size of 1000 bp was produced by four (12.5%) of the isolates.

**Conclusion:**

The presence of these genes with a wide degree of polymorphism confirmed the pathogenic potential of *S. aureus* and their association with clinical manifestations in mastitis among cattle and buffalo.

## Introduction

Protein A of *Staphylococcus aureus*, one of the important virulence factors encoded by *spa* gene is a surface protein that binds to the IgG molecules and evades phagocytosis thus in turn contributing to the development of the disease [1,2]. The *spa* gene is composed of functionally distinct regions, i.e. Fc binding region, X-region and at C-terminus. The X-region of the *spa* gene includes a variable number of 24-bp repeats [3,4] and because of this the *spa* genes have been the most widely used markers for molecular typing. Polymerase chain reaction-restricted fragment length polymorphism (PCR-RFLP) studies of these genes were found to be quite useful in typing *S. aureus* strain and have been considered good in regards to typability, reproducibility and discriminatory power [5]. The strains with more than seven repeats in the X-region have also been considered to be pathogenic[6].

Clumping factor is an important adhesion protein of *S. aureus* that is governed by *clf*A gene [6] and is thought to be essential for colonization and establishment of infections. It participates in the infection process by facilitating bacterial binding via soluble or immobilized fibrinogen as fibrinogen plays a significant role in platelet thrombus formation [7]. Clumping factor has been reported to be present in the majority of isolates [8] and the gene *clf*A has been reported to be important in pathogenicity of bovine mastitis [6]. Tuchscherr *et al*. [9] showed that antibodies to *clf*A enhanced the protection against infection provided by capsular polysaccharides antibodies.

The present work reports *spa* (X-region) and *clf*A gene typing in *S. aureus* obtained from milk from cattle and buffalo with clinical mastitis.

## Materials and Methods

### Ethical approval

All the procedures have been carried out in accordance with the guidelines laid down by the Institutional Ethics Committee and in accordance with local laws and regulations.

### Isolation of *S. aureus*

In the present investigation, 32 *S. aureus* (16 each from cattle and buffalo) were isolated from mastitic milk and identified as per standard procedures [10]. All phenotypically identified isolates were further confirmed by 23S rRNA ribotyping [11].

### Amplification of *spa* and *clf*A gene

Amplification of the *spa* gene was done as described by Frenay *et al*. [12] using 5’CAAGCACCAAAAGAGGAA3’ (F) and 5’CACCAGGTTTAACGACAT3’ (R) primers. Briefly, the reaction mixture of 30μl was prepared by mixing 20.4 μl deionised water, 2.5 μl10x Buffer, 1.8 μl MgCl_2,_ 1.0 μl Primer-1 (10 pM/μl), 1.0 μl Primer-2 (10 pM/μl), 0.6μl dNTP-mix (10 mM), 0.2 μl *Taq* DNA polymerase (5 U/μl) and 2.5 μl template DNA (25ng/μl). Amplification was carried out in a Veriti thermal cycler (Applied biosystem) as follows: initial 34 cycle of amplification (denaturation at 94°C for 60 s, primer annealing at 55°C for 60 s and primer extension at 70°C for 60 s), and final extension at 72°C for 5 min.

Amplification of the *clf*A gene was done as described by Stephan *et al*. [6] using 5’GGCTTCAGTGCTTGTAGG3’ (F) and 5’TTTTCAGGGTCAATATAAG 3’ (R) primers. Briefly, the reaction mixture of 30μl was prepared by mixing 21.4 μl deionised water, 3.0 μl 10x Buffer, 1.8 μl MgCl_2,_ 1.0μl Primer-1 (10 pM/μl), 1.0 μl Primer-2 (10 pM/μl), 0.6μl dNTP-mix (10 mM), 0.2 μl *Taq* DNA polymerase (5U/μl) and 1.0 μl template DNA (25ng/μl). Amplification was carried out in a Veriti thermal cycler (Applied biosystem) as follows: Initial 34 cycle of amplification (denaturation at 94°C for 60 s, primer annealing at 55°C for 60 s and primer extension at 70°C for 60 s), and final extension at 72°C for 5 min. The PCR products, after addition of 2 μl of tracking dye were resolved in 1.2% agarose gels prepared in ×1.0 TBE buffer containing 0.5 μg/ml of ethidium bromide. 50 bp and 100 bp DNA ladder were used as molecular marker. The amplification products were electrophoresed for 50-60 min at 100 Volts. The gel was then visualized under gel documentation system (ENDURO GDS).

## Results

The ribotyping produced an amplicon of 1250 bp in 32 isolates confirming them to be *S. aureus*.

### Amplification of *spa*-X region gene

**Figure-1:**
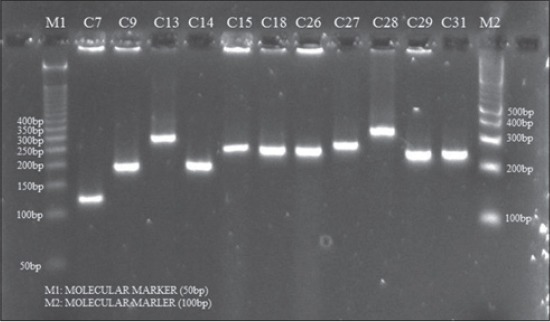
Agarose gel electrophoresis of amplicons of *spa* gene (X-region) of *S. aureus* isolates obtained from cattle mastitic milk

**Figure-2:**
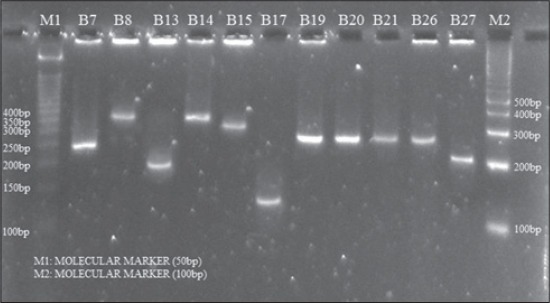
Agarose gel electrophoresis of amplicons of *spa* gene (X-region) of *S. aureus* isolates obtained from buffalo mastitic milk

Protein A, encoded by the *spa* gene, is one of the virulence factors involved in the staphylococcal pathogenesis. The amplification of X region of the *spa* gene produces amplicons of variable sizes depending on the number of 24 bp tandem repeats and this property of the organisms is being used for differentiation among various isolates by the scientists [6,13-15]. The number of repeats in the X region of the *spa* gene correlates with the virulence level of the strains. In the present investigation all the isolates from both cattle and buffalo produced *spa* amplicons of seven types ranging between 120 and 380 bp (Figures-1 and 2). The *spa* gene X-region amplicons produced by cattle isolates were of greater variability than that of isolates from buffalo. The cattle isolates produced seven different types of *spa* amplicons *viz*. 120, 150, 200, 250, 280, 300, and 330 bp with calculated number of repeats of 3, 4, 6, 8, 10, 11 and 12, respectively (Table-1). The buffalo isolates produced 5 types of *spa* amplicons the sizes of which were 150, 200, 250, 330 and 380 with calculated number of repeats of 5, 7, 9, 12, and 14, respectively. The amplicon of 250 bp was produced by maximum numbers of isolates (Table-2).

**Table-1:** *spa* gene (X- region) polymorphism in *S. aureus* isolates from cattle with clinical mastitis.

Isolate numbers	Total isolates	*Spa* gene amplicon (bp)	Total number of repeats
C37	1	120	3
C7	1	150	4
C9, C14, C23	3	200	6
C15, C18, C26, C29, C31, C38	6	250	8
C27, C36	2	280	10
C13	1	300	11
C24, C28	2	330	12

S. aureus=Staphylococcus aureus

**Table-2:** *spa* gene (X- region) polymorphism in *S. aureus* isolates from buffalo with clinical mastitis.

Isolate numbers	Total isolates	*Spa* gene amplicon (bp)	Total number of repeats
B17	1	150	4
B13, B27	1	200	6
B3, B7, B19, B20, B21 B26, B37, B38, B43	9	250	8
B15, B30	2	330	12
B8, B14	2	380	14

S. aureus=Staphylococcus aureus

### Amplification of *clf*A gene

**Figure-3:**
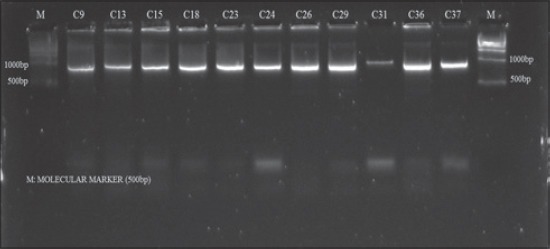
Agarose gel electrophoresis of amplicons of *clf*A gene of *S. aureus* isolates obtained from cattle mastitic milk

**Figure-4:**
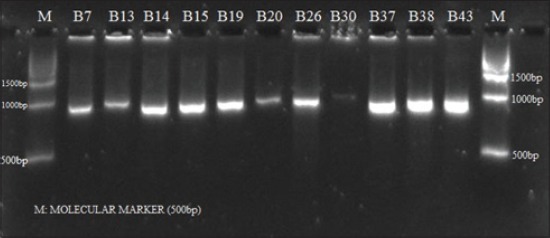
Agarose gel electrophoresis of amplicons of *clf*A gene of *S. aureus* isolates obtained from buffalo mastitic milk

Clumping factor A (*clf*A) is a cell surface-associated protein of *S. aureus* that promotes binding of this pathogen to both soluble and immobilized fibrinogen and thought to be essential for colonization. Gene *clf*A has been reported to be important in pathogenicity of bovine mastitis [6]. In the present investigation, *clf*A gene was amplified in 27 out of 32 isolates from cattle and buffalo producing two different amplicons of 900 and 1000 bp sizes showing polymorphism (Figures-3 and 4). The most (71.80%) of the isolates produced amplicons of 900 bp while amplicon size of 1000 bp was produced by four (12.5%) of the isolates. The remaining five (15.6%) isolates were considered *clf*A deficient.

## Discussion

### Amplification of *Spa* (X-region) gene

Our observations were in complete agreement with many workers such as Salasia *et al*. [8] who obtained nine different sized amplicons of 100, 150, 200, 230, 240, 250, 270, 290 and 340 bp in *S. aureus* isolates from bovine subclinical mastitis, Annemuller *et al*. [16] who obtained amplified fragments of 120, 150, 170, 250 and 300 bp with calculated number of repeats of 3, 4, 5, 8 and 10 respectively and Indrajulianto [17] who studied X-region of protein-A yielding amplicons of variable size *viz*. 100, 150, 200, 250, 280, 300 and 330 bp.

Marques *et al*. [18] reported presence of *spa* gene in all of the isolates from bovine mastitis, showing variable amplicon sizes with 300 bp being the prevalent size. Contrary to the results in the present study, uniform amplicons of 300 bp size were obtained by Suleiman *et al*. [19] in 20 isolates of *S. aureus* from subclinical bovine mastitis. The *S. aureus* isolates have also been reported to be *spa* gene-deficient by Shakeri *et al*. [20].

In the present study, 24 isolates were considered pathogenic since they possessed more than seven repeats. On the other hand, no correlation was reported between tandem repeats and pathogenicity of the isolates by Nashev *et al*. [21] from humans, Kuzma *et al*. [4] and Jakubczak *et al*. [22] in isolates from mastitc cows and Kurlenda *et al*. [23] in human isolates.

### Amplification of *clf*A gene

Our results were in complete agreement with Karahan *et al*. [7] who reported 84 (91.3%) out of 92 *S. aureus* isolates to show presence of *clf*A with two different amplicons at the molecular length of approximately 900 bp and1000 bp. Similarly, Memon *et al*. [24] also reported that the size of *clf*A gene amplicon varied from 900 bp to 1000 bp in *S. aureus* isolates associated with bovine mastitis. Among the *S. aureus* strains representing the clinical mastitic cow, 13 strains (54.2%) had an amplicon size of 900 bp, while 11 strains (45.8%) had an amplicon size of 1000 bp and the *S. aureus* strains obtained from sub-clinical mastitis 2 (12.5%) strains had a *clf*A amplicon size of 900 bp and 14 (87.5%) strains an amplicon size of 1000 bp [25].

On the contrary to our observations, polymorphism was not reported for this gene by many workers. Stephan *et al*. [6] recorded amplicon with a size of approximately 980 bp for all 34 *S. aureus*. Likewise, other workers [26-28] also found amplicons of same sizes with no polymorphism. Akineden *et al*. [2] and Salasia *et al*. [8] reported amplification of the clumping factor gene *clf*A resulting in a single amplicon with a size of approximately 1000 bp from *S. aureus*. Similarly, Kalorey *et al*. [29] characterized 37 strains of *S. aureus* of bovine milk origin for *clf*A gene and obtained an amplicon of approximately 1042 bp only. Likewise, Moroni *et al*. [30] characterized *S. aureus* isolated from milk from chronically infected alpine dairy goats and obtained a *clf*A amplicon of 1030 bp in all 28 isolates. Nathawat [31] also reported amplicons of 1050 bp in isolates from the goat mastitis from same study area. In study of Salem-Bekhit *et al*. [27] also, amplification of the clumping factor (*clf*A) gene resulted in a single amplicon with a size of approximately 985 bp for all 68 *S. aureus* strains indicating no size polymorphisms of this gene.

## Conclusion

The present study revealed presence of *spa* and *clf*A gene with a wide degree of polymorphism. It directly correlates with the pathogenic potential of an organism and their association with clinical manifestations in mastitis among cattle and buffalo. In the present study, 24 isolates were considered pathogenic since they possessed more than seven repeats. The number of repeats along the X region of the *spa* gene correlates with the virulence level of the strains. Likewise, *clf*A gene polymorphism was also recorded among isolates.

## Authors’ Contributions

RY and AKK planned and designed the study. RY and SKK conduct the experiment. Lab analysis was carried out by RY and JY. Writing of the manuscript is done by RY and JY under the guidance of AKK. All authors read and approved the final manuscript.
